# Multi-omics-based investigation of *Bifidobacterium’s* inhibitory effect on glioma: regulation of tumor and gut microbiota, and MEK/ERK cascade

**DOI:** 10.3389/fmicb.2024.1344284

**Published:** 2024-04-18

**Authors:** Huali Fan, Yuhan Wang, Mingyu Han, Li Wang, Xue Li, Xi Kuang, Junrong Du, Fu Peng

**Affiliations:** ^1^Key Laboratory of Drug-Targeting and Drug Delivery System of the Education Ministry, Sichuan Engineering Laboratory for Plant-Sourced Drug and Sichuan Research Center for Drug Precision Industrial Technology, Department of Pharmacology, West China School of Pharmacy, Sichuan University, Chengdu, China; ^2^Jiangsu Sanshu Biotechnology Co., Ltd., Nantong, China

**Keywords:** glioma, *Bifidobacterium*, tumor microbiota, gut microbiota, MEK/ERK cascade, serum metabolites

## Abstract

Glioma, the most prevalent primary tumor of the central nervous system, is characterized by a poor prognosis and a high recurrence rate. The interplay between microbes, such as gut and tumor microbiota, and the host has underscored the significant impact of microorganisms on disease progression. *Bifidobacterium*, a beneficial bacterial strain found in the human and animal intestines, exhibits inhibitory effects against various diseases. However, the existing body of evidence pertaining to the influence of *Bifidobacterium* on glioma remains insufficient. Here, we found that *Bifidobacterium* reduces tumor volume and prolongs survival time in an orthotopic mouse model of glioma. Experiments elucidated that *Bifidobacterium* suppresses the MEK/ERK cascade. Additionally, we noted an increase in the α-diversity of the tumor microbiota, along with an augmented relative abundance of *Bifidobacterium* in the gut microbiota. This rise in *Bifidobacterium* levels within the intestine may be attributed to a concurrent increase in *Bifidobacterium* within the glioma. Additionally, *Bifidobacterium* induced alterations in serum metabolites, particularly those comprised of organonitrogen compounds. Thus, our findings showed that *Bifidobacterium* can suppress glioma growth by inhibiting the MEK/ERK cascade and regulating tumor, and gut microbiota, and serum metabolites in mice, indicating the promising therapeutic prospects of *Bifidobacterium* against glioma.

## 1 Introduction

Gliomas, originating from neuroglial stem or progenitor cells, are the most prevalent primary heterogeneous tumors in the central nervous system (CNS). These include glioblastoma (GBM), astrocytoma, oligodendroglioma, and ependymal tumors (Weller et al., 2015). In the United States, GBM is the most prevalent malignant brain tumor, accounting for 14.2% of all tumors and 50.1% of all malignant tumors (Ostrom et al., 2022). In China, the incidence rate of glioma ranges from 5 to 8 per 100,000, with a 5-year mortality rate second only to pancreatic cancer and lung cancer among systemic tumors (National Health Commission of the People’s Republic of China, 2022). The fifth edition of the World Health Organization Classification of Tumors of the CNS employs Arabic numerals to categorize gliomas into grades 1 through 4, based on their histological and molecular characteristics (Reuss, 2023). This new fifth edition has redefined many tumors, including adult-type diffuse glioma, pediatric-type diffuse low-grade and high-grade glioma, and limited astrocytic glioma, as a “superclass” (Smith et al., 2022). The incidence rate of glioma varies based on race/ethnicity, sex, and age (Dubrow and Darefsky, 2011). In clinical settings, the treatment for glioma primarily involves surgical intervention combined with chemotherapy or radiotherapy. Temozolomide and the PCV regimen (procarbazine, lomustine, and vincristine) serve as the mainstay chemotherapy options (National Health Commission of the People’s Republic of China, 2022). Unfortunately, there is an urgent need to find more effective treatment for glioma due to the emergence of drug resistance, poor prognosis, and high recurrence rate.

The historical connection between cancer and microbiota dates back as far as 4,000 years, with documented instances of spontaneous tumor regression in patients infected with *Streptococcus pyogenes* in 1868 (Sepich-Poore et al., 2021). Although approximately 1,012 microbial species exist on Earth, only 11 have been classified as “carcinogenic microbes” by the International Association for Cancer Registries (IARC Working Group on the Evaluation of Carcinogenic Risks to Humans, 2012; Locey and Lennon, 2016). A study reported the presence of microbes in various tumors, including breast cancer and glioma, each exhibiting distinct compositions and content (Nejman et al., 2020). Tumor microbiota may originate from the gut microbiota breaching the mucosal barrier to infiltrate tumor tissue, from normal adjacent tumor tissues, or from microbes circulating in the bloodstream (Xie et al., 2022). Due to the tumor-type- and subtype-specific nature of tumor microbiota, these microbes may serve as diagnostic tools. Microbes attracted to tumors could be harnessed as precise vectors for anticancer drug delivery, and the unique microbiota composition in different patient survival profiles holds promise as a potential prognostic tool (Ma et al., 2021). Tumor microbiota plays a dual role: it can, on the one hand, facilitate cancer metastasis in spontaneous breast cancer (Fu A. et al., 2022), and, on the other hand, deliver specific bacteria to tumors to promote tumor cell pyroptosis (Liu et al., 2022).

Numerous studies have demonstrated a close association between the intestinal microbiota dysbiosis and tumorigenesis. The gut microbiota not only affects the development of tumors but also exerts an impact on the efficacy of tumor therapies (Zhao et al., 2023). *Bifidobacterium*, belonging to the *Actinobacteria* phylum, constitutes a crucial core and beneficial microbe in the intestine flora of humans and animals (Ngo et al., 2019). *Bifidobacterium* confers various beneficial effects, including promoting biotransformation, outcompeting pathogens, regulating oncogenic gene expression, and maintaining immune homeostasis (Wei et al., 2018). When combined with *Lactobacillus, Bifidobacterium* enhances cell apoptosis by activating pro-caspases, downregulating the anti-apoptotic factor B-cell lymphoma-2 (Bcl-2), and upregulating the pro-apoptotic factor BCL-2-associated X (Bax) protein (Nowak et al., 2019). Furthermore, *Bifidobacterium* enhances the anti-tumor effects of Programmed cell death 1 ligand 1 (PD-L1) inhibitors, independent of tumor immune antigens, *in vivo* (Sivan et al., 2015). In tumor-bearing mice in which CD47 inhibitors are ineffective, injection of *Bifidobacterium* resulted in a response to anti-D47 treatment In cases where CD47 inhibitors are ineffective in tumor-bearing mice, the administration of *Bifidobacterium* results in a positive response to anti-D47 treatment (Shi et al., 2020).

This is the first study to investigate the impact of *Bifidobacterium* on glioma growth in the GL261 orthotopic mouse model, employing gavage treatment with mixtures of *Bifidobacterium breve*, *Bifidobacterium longum*, *Bifidobacterium lactis*, and *Bifidobacterium bifidum*. Our findings demonstrate that *Bifidobacterium* inhibits glioma progression, in part, by modulating the MEK/ERK cascade, gut microbiota, tumor microbiota, and serum metabolites. Thus, these results suggest that *Bifidobacterium* may hold promise as a potential therapeutic agent against glioma.

## 2 Materials and methods

### 2.1 Cell culture

Cell line GL261 was bought from Hunan Fenghui Biotechnology Co. Ltd., China. and cultured in DMEM media (Gibco, MA, United States) containing 10% fetal bovine serum (Gibco, MA, United States) and 1% penicillin/streptomycin (Invitrogen, MA, United States) at 37°C, 5%CO_2_.

### 2.2 Bacterial preparation

*B. breve* (BBR-15), *B. longum* (JBLC-141), *B. lactis* (JYBR-190), and *B. bifidum* (JYBB-163) freeze-dried powders were procured from Shandong Zhongke Jiayi Biological Engineering Co. Ltd., China. A concentration of 4 × 10^9^ CFU/0.4 mL (equivalent to 1 × 10^9^ CFU for each *Bifidobacterium*) was attained by suspending These four types of *Bifidobacterium* in sterile saline (Wang et al., 2022). The bacterial identification results are presented in Supplementary Tables S1–S5 (Supplementary sequences S1–S4).

### 2.3 Animal experiment design

Female C57BL/6 mice (4 weeks old) were procured from Chengdu Dashuo Laboratory Animal Company (Chengdu, China) and were maintained at a temperature of 22°C ± 2°C under a 12 h:12 h light–dark cycle within specific pathogen-free facilities. All animal experiments adhered to the ethical regulations stipulated by the Experimental Animal Administration of Sichuan University. Three sets of C57BL/6 mice were employed in this study: The first set was allocated for the construction of a survival curve (*n* = 6), the second set for indicator detection (*n* = 12), and the third set for the investigation of liver and kidney function (*n* = 8).

For survival curve analysis, the first set of mice were distributed into groups at random: the Model (Mod) group and the Model-*Bifidobacterium* (Mod-BIF) group. The Mod-BIF group received daily gavages of a *Bifidobacterium* mixture at a dose of 4 × 10^9^ CFU/400 μL (with each dose containing 1 × 10^9^ CFU) from day −14 to day 30 (Wang et al., 2022). The Mod group received a daily equal-volume saline solution from day −14 to day 30. For tumor induction, anesthetized mice of both groups were stereotactically injected with 1 × 10^5^ GL261 cells (1 μL) into the right striatum at a rate of 0.4 μL/min on day 0. The stereotaxic coordinates with respect to the bregma were as follows: 1.0 mm anterior, +1.5 mm lateral, and 3.5 mm ventral (Lam et al., 2018).

For indicator detection, the second set of mice were distributed into groups at random: the Mod group and the Mod-BIF group. The Mod-BIF group received daily gavages of a *Bifidobacterium* mixture at a dose of 4 × 10^9^ CFU/400 μL (with each dose containing 1 × 10^9^ CFU) from day −14 to day 30. The Mod group received a daily equal-volume saline solution from day −14 to day 30. For tumor induction, anesthetized mice of both groups were stereotactically injected with 1 × 10^5^ GL261 cells (1 μL) into the right striatum at a rate of 0.4 μL/min on day 0. The stereotaxic coordinates with respect to the bregma were as follows: 1.0 mm anterior, +1.5 mm lateral, and 3.5 mm ventral. The mice were euthanized 30 days after the inoculation.

For the investigation of liver and kidney function, the third set mice were distributed into groups at random: the control (Con) group, the Mod group and the Mod-BIF group. The Mod-BIF group received daily gavages of a *Bifidobacterium* mixture at a dose of 4 × 10^9^ CFU/400 μL (with each dose containing 1 × 10^9^ CFU) from day −14 to day 30. The Mod group received a daily equal-volume saline solution from day −14 to day 30. For tumor induction, anesthetized mice of the Mod and Mod-BIF groups were stereotactically injected with 1 × 10^5^ GL261 cells (1 μL) into the right striatum at a rate of 0.4 μL/min on day 0. The stereotaxic coordinates with respect to the bregma were as follows: 1.0 mm anterior, +1.5 mm lateral, and 3.5 mm ventral. The mice were euthanized 30 days after the inoculation. The mice of the Con group did not receive any treatment.

### 2.4 Magnetic resonance imaging

Cerebral MRI was conducted *in vivo* on a 7 T MRI scanner designed for small animals (Bruker BioSpec 70/30, Germany). 2% isoflurane was inhaled to induce anesthesia in all of the mice. Imaging was performed utilizing a 2D T2-weighted sequence in the axial orientation with the following parameters: repetition time = 2,625 ms, echo time = 33 ms, number of averages = 4, matrix = 256 × 256, and slice thickness = 0.6 mm. The ITK-SNAP program was utilized to calculate tumor volumes.

### 2.5 Fecal samples and tissue collection

Fresh fecal pellets were collected and immediately frozen at −80°C on day 30 post-inoculation. Following the experiments, 1% pentobarbital sodium (40 mg/kg) solution was used to anesthetize all mouse. All mice were subsequently subjected to intracardial perfusion with cold, sterilized saline. Tissues from the brain, liver, kidney, ileum, and colon were harvested. Some portions of these tissues were snap-frozen in liquid nitrogen and stored at −80°C, while others were fixed using a 4% paraformaldehyde solution and subsequently embedded in paraffin.

### 2.6 Measurement of serum AST, ALT, BUN, and Cr

Serum was obtained from mice by centrifugation at 3,500 rpm for 10 min, following a 30-min incubation in a 37°C oven. Serum levels of AST, ALT, BUN, and Cr were assessed using respective kits as per the manufacturer’s instructions (Nanjing Jiancheng Bioengineering Institute, China).

### 2.7 Histological and immunohistochemical analyses

The paraffin-embedded tissues were sectioned into 3-μm-thick slides. According to standard histological protocols, hematoxylin and eosin (HE) staining was conducted. For immunohistochemistry (IHC), the following primary antibodies were used to incubate the slides, respectively, after blocking: bacterial lipopolysaccharide (LPS; HycultBiotech #HM6011, Netherlands), lipoteichoic acid (LTA; Santa Cruz, #sc-57752, United States), occludin (Servicebio #GB111401, China), and zonula occludens 1 (ZO-1; Servicebio #GB111981, China). IHC quantification involved the calculation of positive areas (occludin and ZO-1) using Aipathwell software (Version 2.0, Servicebio, China).

### 2.8 Reverse transcription quantitative real-time PCR

The total RNA was isolated using TRIzol reagent (Thermo Fisher Scientific, MA, United States) and subsequently reverse-transcribed using the RevertAid First Strand cDNA Synthesis Kit (Thermo Fisher Scientific). Real-time polymerase chain reaction was conducted according to established protocols from prior studies, and data were analyzed utilizing the 2^−ΔΔCT^ method. Primer sequences for the target genes are listed in Table 1.

**Table 1:** PCR primer sequences.

Gene	Forward (5′-3′)	Reverse (3′-5′)
Wnt5a	GGAACGAATCCACGCTAAGGGT	AGCACGTCTTGAGGCTACAGGA
Sdha	GGAACACTCCAAAAACAGACCT	CCACCACTGGGTATTGAGTAGAA

### 2.9 Western blot

Total proteins were extracted using RIPA buffer (Beyotime, Shanghai, China) according to the manufacturer’s instructions. The protein lysate was subjected to electrophoresis and transferred onto a PVDF membrane (Bio-Rad, CA, United States). The following primary antibodies sourced from CST (Shanghai, China): p-MEK (Ser217/221; 1:1000; 9154), MEK (1:1000; 8727), p-ERK1/2 (Thr202/Tyr204; 1:2000; 4370), and ERK (1:1000; 4695), PD-1 (1:1000; 84651), PD-L1 (1:1000; 60475), and p-NF-κB (1:1000; bs-0982R) from Bioss (Beijing, China), NF-κB (1:1000; sc-8008) from Santa Cruz Biotechnology (Texas, United States) were used to incubate the membrane, respectively. The proteins were detected using ECL Plus Reagent (Beyotime) and quantified using Image Lab software.

### 2.10 Quantitative RNA sequencing

RNA-seq analysis was performed by LC-Bio (Hangzhou, China). Briefly, glioma tissues were collected and processed using TRIzol reagent. Utilizing UMI technology, each sequence fragment was tagged with unique identifiers, thereby mitigating the impact of PCR-induced duplications on transcriptome quantification accuracy. The RNA-seq reads were aligned to the mouse genome (GRCh37/hg19) using the Hisat2 software (version 2.0.4). Transcript abundance was quantified in terms of fragments per kilobase of exon per million fragments. Significantly differential expression was defined with a threshold of *p* < 0.05 and |log2(fold change)| ≥ 1 The raw sequence data have been submitted to the NCBI Gene Expression Omnibus (GEO) with accession number GSE246028.

### 2.11 Tumor microbial analysis

The frozen glioma samples were processed using the CTAB method. Subsequently, the 16S rRNA gene was amplified and sequenced, involving the simultaneous amplification of five regions of the 16S rRNA gene. The resulting libraries were sequenced using the Illumina NovaSeq 6000 system. Reads were demultiplexed per sample, filtered, and aligned to the five amplified regions according to the primer sequences. To generate coherent profiling results, the Short MUlitiple Regions Framework method was employed, addressing a maximum likelihood problem by combining read counts from the five regions. Reference data were obtained from the GreenGenes database (May 2013 version, with some improvements). The raw sequence data have been submitted to the NCBI Short Read Archive (SRA) with accession number PRJNA1021034.

### 2.12 Gut microbial analysis

DNA from fecal samples was extracted following the manufacturer’s CTAB instructions. The bacterial and archaeal 16S rRNA gene’s V3-V4 region was amplified using the 341F primer (5′-CCTACGGGNGGCWGCAG-3′) and the 805R primer (5′-GACTACHVGGGTATCTAATCC-3′).

The 16S rRNA amplicons were sequenced on an Illumina NovaSeq platform following LC-Bio’s instructions. Paired-end reads were merged with FLASH. Raw reads underwent quality filtering, applying specific filtering conditions to obtain high-quality clean tags using fqtrim (v0.94). Chimeric sequences were removed using Vsearch software (v2.3.4). After dereplication using DADA2, feature tables and feature sequences were generated. α diversity and β diversity were calculated by randomly normalizing the sequences. Feature abundance was normalized using SILVA (release 138) classifier. QIIME2 was employed for α diversity analysis, assessing species diversity complexity. β diversity was calculated using QIIME2, and R packages were utilized to generate corresponding graphs. Sequence alignment was performed via BLAST, and feature sequences were annotated using the SILVA database for each representative sequence. Additional diagrams were generated using the R package (v3.5.2). The raw sequence data have been submitted to the NCBI Short Read Archive (SRA) with accession number PRJNA1021780.

### 2.13 Untargeted metabolomics of mouse serum by LC-MS

The metabolic extracts from serum were analyzed using LC-Bio. Serum samples were collected, and metabolites were extracted with a 50% buffer solution. Pooled quality control samples were prepared by combining 10 μL from each extraction mixture. Chromatographic separations were performed using an ultra-performance liquid chromatography (UPLC) system (SCIEX, UK) with an ACQUITY UPLC T3 column (100 mm*2.1 mm, 1.8 μm, Waters, UK) for reversed-phase separation. The detection of eluted metabolites from the column was performed using a high-resolution tandem mass spectrometer, the Triple TOF5600 Plus (SCIEX). The acquired MS secondary data were subsequently matched with an in-house metabolite standard secondary profile library to identify differential metabolites.

### 2.14 Statistics analysis

Statistical analyses were carried out with the SPSS 26.0 software. Log-rank test was used for Kaplan–Meier survival curves. Except as otherwise indicated, statistical difference between 2 groups was identified Statistical analyses were conducted using SPSS 26.0 software. The log-rank test was applied to Kaplan–Meier survival curves. In cases where not otherwise specified, statistical differences between two groups were determined using an independent sample *t*-test. For multiple comparisons, a one-way analysis of variance (ANOVA) was employed, followed by a Tukey *post-hoc* test. Significance was attributed to differences at *p* < 0.05. In figures, “*” indicated *p* < 0.05, “**” indicated *p* < 0.01.

## 3 Results

### 3.1 *Bifidobacterium* inhibited glioma growth in mice without hepatic or renal toxicity

**Figure 1:**
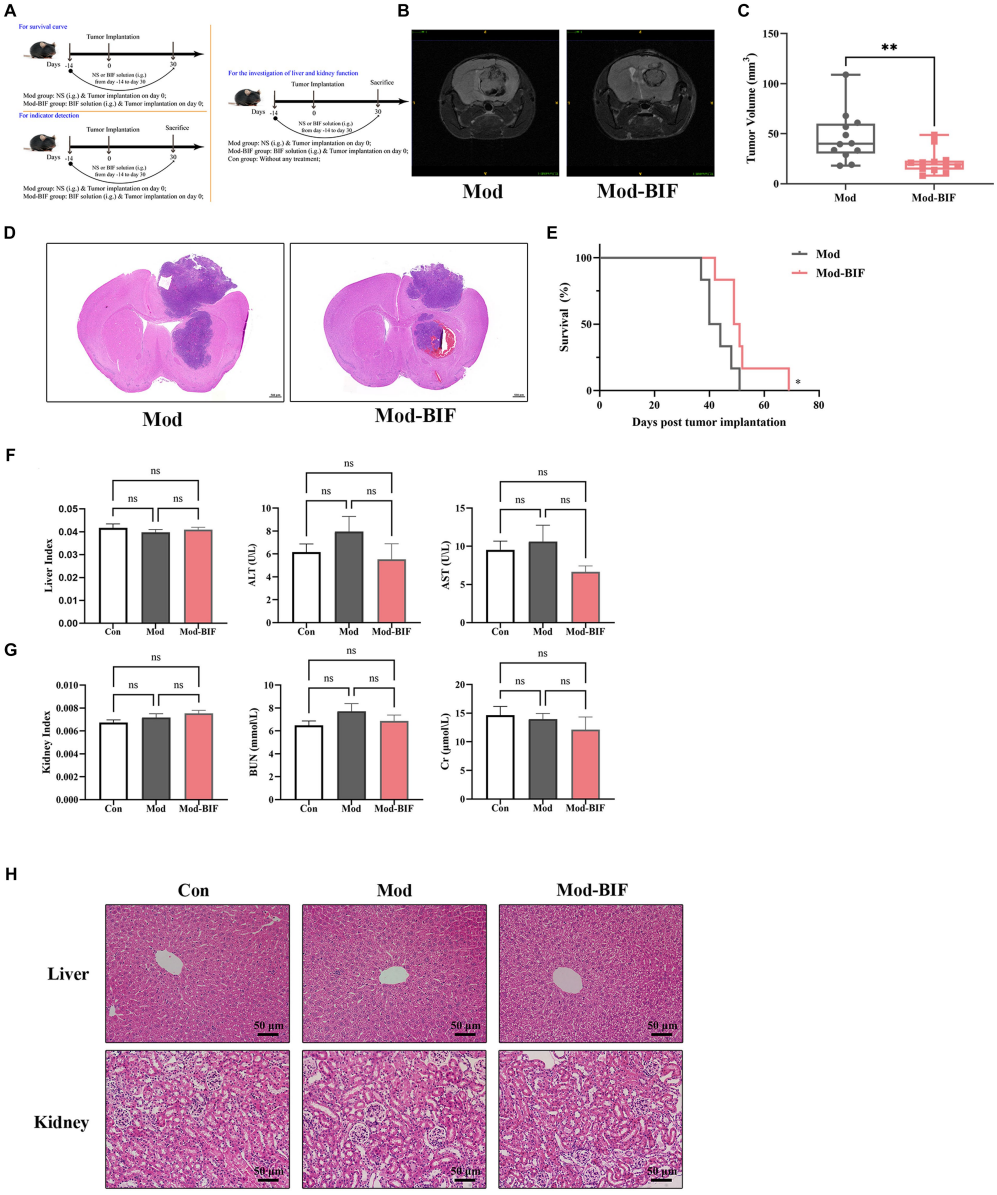
*Bifidobacterium* inhibited glioma without hepatic or renal toxicity. **(A)** Schematic of the experimental timeline. **(B)** Representative T2-weighted MRI images and **(C)** tumor volume quantification of glioma mice receiving either PBS or *Bifidobacterium* at day 30 post-tumor implantation (*n* = 12; independent sample *t*-test). **(D)** Representative HE-stained coronal brain sections. **(E)** Kaplan–Meier survival curves (*n* = 6; log-rank test). **(F)** Liver index, serum levels of ALT and AST. **(G)** Kidney index, serum levels of BUN and Cr. **(H)** Representative HE-stained liver and kidney sections showing the safety of *Bifidobacterium* (*n* = 8; ANOVA). Mod, Model; BIF, *Bifidobacterium*; NS, normal saline. Experimental data are expressed as the mean ± SEM. ^*^p < 0.05, ^**^p < 0.01.

To ascertain the effectiveness of *Bifidobacterium* in inhibiting glioma growth in mice, we administered the Mod-BIF group with 400 μL of *Bifidobacterium* solution (4 × 10^9^ CFU) daily through intragastric gavage from day −14 to day 30. On day 0, mice of the Mod and Mod-BIF groups were implanted with 1 × 10^5^ GL261 cells into the right striatum (Figure 1A). Subsequently, we employed cerebral T2-weighted MRI sequences to evaluate tumor volume on day 30 (Figure 1B). The results indicated a significant reduction in tumor volume in the Mod-BIF group compared with the Mod group (46.35 ± 16.34 mm^3^ vs. 21.71 ± 10.75 mm^3^, *p* < 0.01; Figure 1C). This observation was further supported by HE staining of glioma tissues (Figure 1D). As depicted in Figure 1E, the administration of *Bifidobacterium* resulted in a slight extension of median survival time in mice with glioma (42 days vs. 52 days, *p* < 0.05; Figure 1E). To delve deeper into the effect of *Bifidobacterium* on liver and kidney function, we assessed the organ indices of the liver and kidney, as well as the serum concentrations of ALT, AST, BUN, and Cr, alongside HE staining of liver and kidney of mice. The results showed no significant differences in the organ indices of the liver and kidney, nor in the levels of serum ALT, AST, BUN, and Cr among the three groups of mice (Figures 1F,G). Moreover, HE staining indicated the absence of noticeable lesions in the liver and kidneys of the mice (Figure 1H). Therefore, it can be concluded that *Bifidobacterium* effectively inhibits glioma growth without causing hepatic or renal toxicity.

### 3.2 *Bifidobacterium* altered the tumor microbiota composition in glioma mice

**Figure 2:**
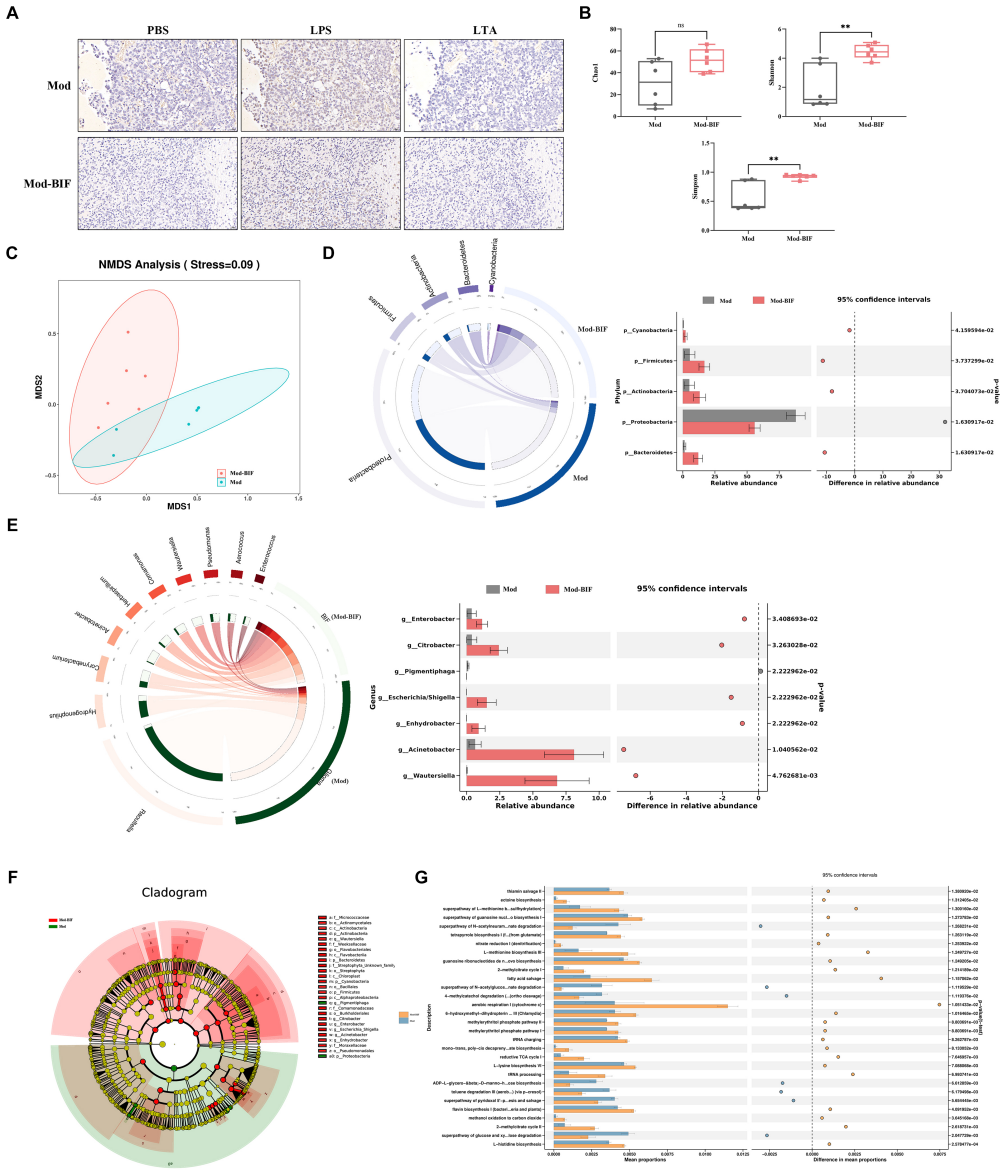
*Bifidobacterium* could regulate intra-tumoral microbiota in glioma mice. **(A)** Slices from tumor tissues of glioma mice were stained with an antibody against bacterial LPS or LTA or PBS to mitigate non-specific staining (×400). **(B)** Comparison of α-diversity of tumor microbiota, including Chao1, Shannon, and Simpson indices in the Mod and Mod-BIF groups. Mann–Whitney U test. **(C)** NMDS analysis based on Bray–Curtis distance. **(D)** Circos of sample and top 5 phylum. A visual circle diagram describing the correspondence between samples and phylum, and the analysis of variance between groups at the phylum level. **(E)** Circos of sample and top 10 genus, and the analysis of variance between groups at the genus level. **(F)** Cladogram of LEfSe analysis between the Mod and Mod-BIF groups. **(G)** Prediction of bacterial metabolite pathway based on PICURSt2 analysis. Mod, Model; BIF, *Bifidobacterium*. Experimental data are expressed as the mean ± SEM, ^**^p < 0.01.

To ascertain the presence of microbiota within tumor tissues, we conducted IHC, utilizing antibodies specific to bacterial LPS and LTA while employing PBS to mitigate non-specific staining. As shown in Figure 2A, bacterial LPS was detected in both the Mod and Mod-BIF groups, whereas LTA showed negative results, consistent with findings from Nejman et al. (2020). These results collectively corroborated the existence of microbiota in gliomas. Subsequently, we conducted 16S rDNA 5R sequencing to investigate alterations in the tumor microbiota in glioma mice. Three metrics (Chao1, Shannon, and Simpson indices) were used to analyze the α-diversity of tumor microbiota. The results showed that *Bifidobacterium* administration led to an elevated Chao1 index trend and a significant increase in both the Shannon and Simpson indices (*p* < 0.01; Figure 2B). Furthermore, nonmetric multidimensional scaling (NMDS) based on Bray–Curtis distance illustrated distinct differences in the composition of tumor microbiota between the Mod and Mod-BIF groups (Figure 2C).

At the phylum level, as shown in Figure 2D, the tumor microbiota in Mod and Mod-BIF groups was primarily dominated by *Proteobacteria*. Compared with the Mod group, the Mod-BIF group exhibited a higher abundance of *Firmicutes*, *Bacteroidetes*, *Cyanobacteria*, and *Actinobacteria*, and a lower abundance of *Proteobacteria*. At the genus level, the Mod group displayed a relatively high abundance of *Raoultella* and *Hydrogenophilus*, while *Corynebacterium*, *Acinetobacter*, and others were relatively abundant in the Mod-BIF group (*p* < 0.05, Figure 2E). Significance analysis revealed that *Enterobacter*, *Citrobacter*, *Acinetobacter*, *Wautersiella*, *Enhydrobacter*, and *Escherichia/Shigella* were significantly more abundant in the Mod-BIF group (*p* < 0.05, Figure 2E). Linear discriminant analysis (LDA) effect size (LEfSe) was employed to further identify specific microbiota, and the results indicated a notable difference in tumor microbiota between the two groups (Figure 2F). Most of the differential bacteria were enriched in the Mod-BIF group, suggesting their potential significance in this group. Furthermore, we utilized Phylogenetic Investigation of Communities by Reconstruction of Unobserved States (PICRUSt) to predict the functional potential of bacterial genes. PICRUSt2, which boasts an expanded reference genome database compared with the PICRUSt1(Langille et al., 2013; Douglas et al., 2019) was employed. As Figure 2G illustrates, the Mod-BIF group exhibited upregulation in several pathways compared with the Mod group, including aerobic respiration I (cytochrome c) and fatty acid salvage (mean proportion > 0.6%).

### 3.3 Effect of *Bifidobacterium* on the intestinal barrier in glioma mice

**Figure 3:**
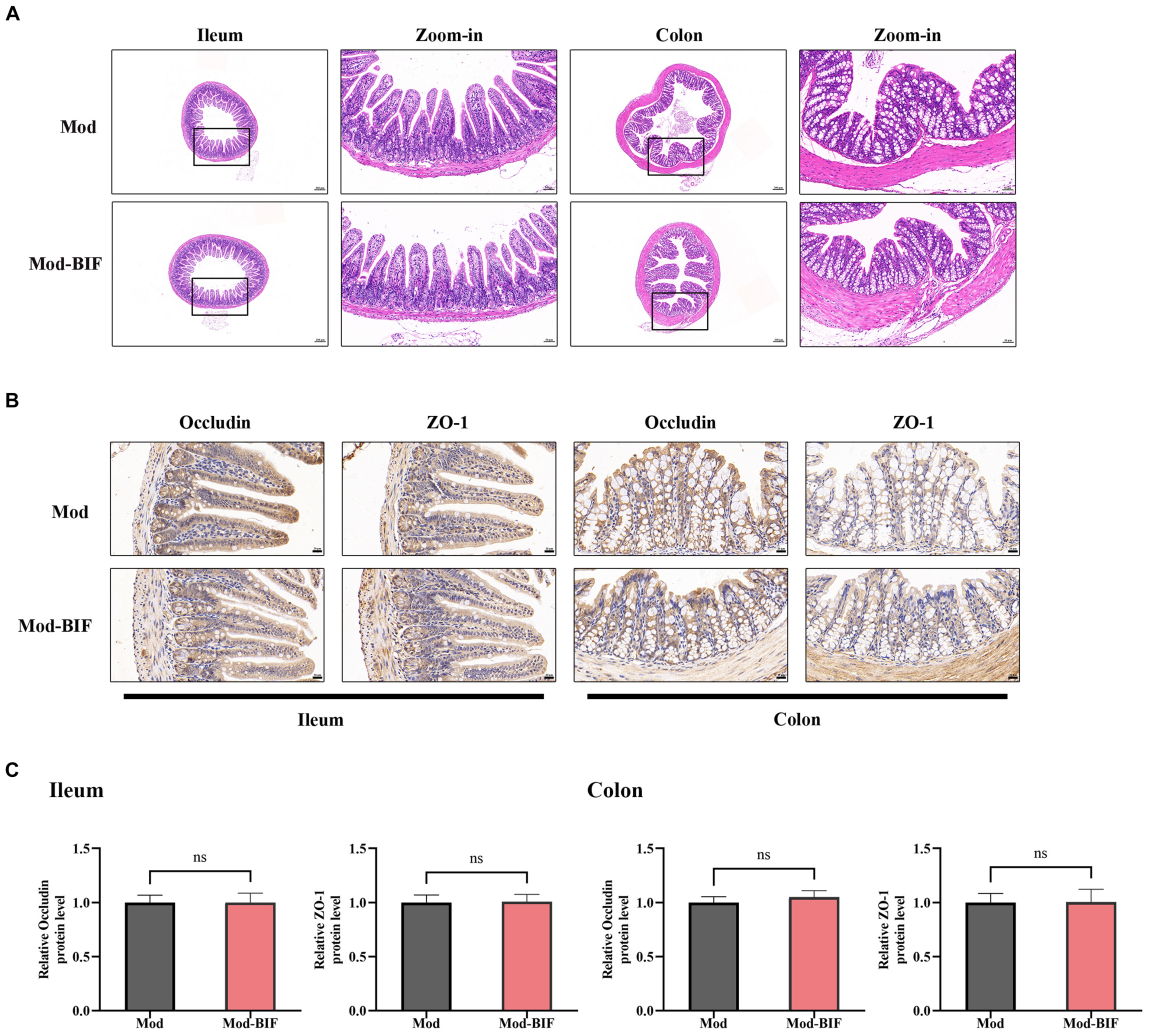
Effect of *Bifidobacterium* on the intestinal barrier in glioma mice Representative **(A)** HE- (×40, ×200), **(B)** occludin-, and ZO-1-stained sections (×400) of ileum and colon tissues from mice. **(C)** Quantification of occludin and ZO-1 immuno-positive areas in ileum and colon tissues (*n* = 6; independent sample *t*-test). Mod, Model; BIF, *Bifidobacterium*. Experimental data are expressed as the mean ± SEM.

The intestinal barrier protects the organism against pathogenic invasions. Its primary physical barrier is upheld by intercellular tight junctions, with occludin, claudin, ZO, and junctional adhesion proteins constituting these junctions (Suzuki, 2020). Prior laboratory investigations have shown that gliomas can induce modest impairment of the intestinal barrier in mice (Wang et al., 2022). Consequently, we aimed to ascertain whether *Bifidobacterium* can ameliorate such damage through HE and IHC staining. As shown in Figure 3A, there were no discernible anomalies observed in the ileum and colon tissues of mice in both experimental groups. Subsequent IHC staining revealed no significant alterations in the protein expression levels of occludin and ZO-1 in the ileum and colon tissues (Figures 3B,C). Thus, to some extent, *Bifidobacterium* may not alleviate the glioma-induced slight damage to the intestinal barrier.

### 3.4 *Bifidobacterium* influenced the composition of gut microbiota in glioma mice

**Figure 4:**
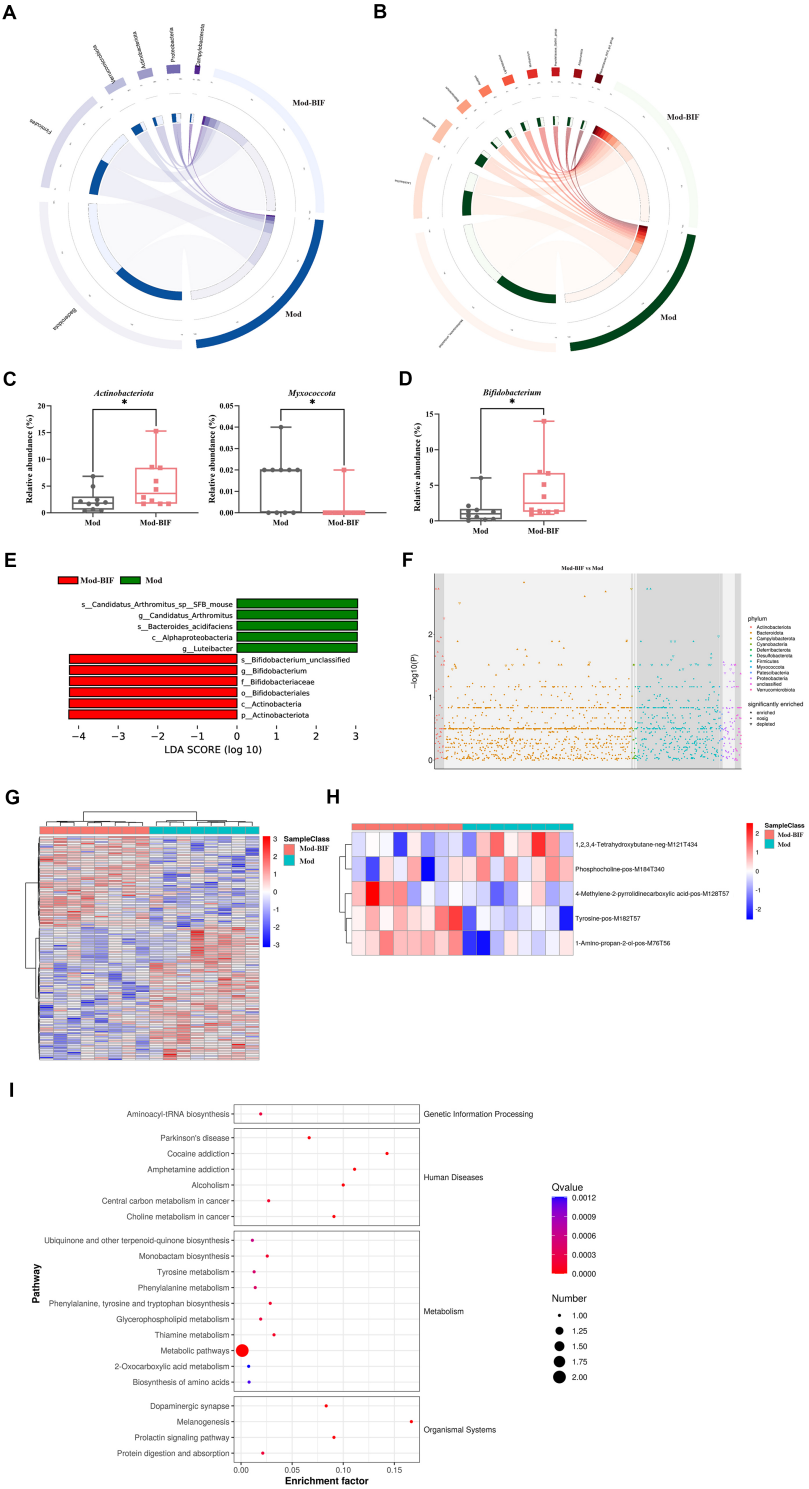
*Bifidobacterium* influenced gut microbiota and serum metabolites in glioma mice. **(A)** Circos of the sample and the top six phylums. **(B)** Circos of the sample and the top 10 genus. **(C)** Relative abundance of *Actinobacteria* and *Myxococcota* in the gut microbiota at the phylum level. **(D)** Relative abundance of *Bifidobacterium* in the gut microbiota at the genus level. **(E)** LDA score of enriched bacterial taxa (|LDA| > 3.0). **(F)** Manhattan plot between the Mod and Mod-BIF groups. **(G)** Differential metabolic ion clustering heatmap (*n* = 8). **(H)** Heatmap of differential metabolites (*n* = 8). **(I)** KEGG pathway enrichment analysis of differential metabolites. Mod, Model; BIF, *Bifidobacterium*; LDA, Linear discriminant analysis. Experimental data are expressed as the mean ± SEM, ^*^p < 0.05.

To compare the differences in gut microbiota composition between the Mod and Mod-BIF groups, we performed 16S rDNA sequencing using fecal samples from mice. The α-diversity of the gut microbiota remained unaffected by *Bifidobacterium* in glioma mice (Supplementary Figure S1A). Furthermore, β-diversity analysis revealed distinctions in the gut microbiota composition between the two groups (Supplementary Figure S1B). At the phylum level in both groups, *Bacteroidetes* and *Firmicutes* were the predominant bacterial taxa (Figure 4A). The Mod-BIF group exhibited higher levels of *Actinobacteriota* and lower levels of *Myxococcota* compared with the Mod group (*p* < 0.05, Figure 4C). Interestingly, upon closer examination at the genus level, administration of *Bifidobacterium* significantly augmented the abundance of *Bifidobacterium* (*p* < 0.05, Figures 4B,D). Similarly, the results from LEfSe indicated that *Bifidobacterium* dominated the microbiota in the Mod-BIF group (Figure 4E). A Manhattan plot further revealed that the administration of *Bifidobacterium* may primarily influence the levels of *Bacteroidetes* and *Firmicutes* in the gut microbiota of glioma mice (Figure 4F).

### 3.5 Effects of *Bifidobacterium* on serum metabolites in glioma mice

Metabolites originating from the gut microbiota can enter the bloodstream and exert regulatory functions in distal organs (Chen et al., 2022). Therefore, we performed an untargeted metabolomic analysis by LC-MS on serum samples collected from mice in both the Mod and Mod-BIF groups. A total of 138 differential metabolic ions were identified, with 56 upregulated ions and 82 downregulated ions meeting the criteria of having a variable importance in projection score of ≥1, a *p* value of <0.05, and a fold change of ≥1.5 or ≤1/1.5 (Figure 4G). Subsequently, we correlated these differential ions to secondary metabolites, resulting in the identification of five differential metabolites (Figure 4H). Among these metabolites, compared with the Mod group, the Mod-BIF group exhibited upregulated 1-amino-propan-2-ol, tyrosine, and 4-methylene-2-pyrrolidinecarboxylic acid levels and downregulated phosphocholine and 1,2,3,4-tetrahydroxybutane levels in the serum. Analysis of these metabolites using the Human Metabolome Database revealed their primary enrichment in organonitrogen compounds (Supplementary Figure S2). Furthermore, results from Kyoto Encyclopedia of Genes and Genomes (KEGG) pathway enrichment demonstrated that these differential metabolites were mainly enriched in metabolic pathways, melanogenesis, thiamine metabolism, and phenylalanine, tyrosine, and tryptophan biosynthesis (Figure 4I).

### 3.6 Combined analysis of metabolomic and gut microbiome as well as gut microbiota and tumor microbiota

**Figure 5:**
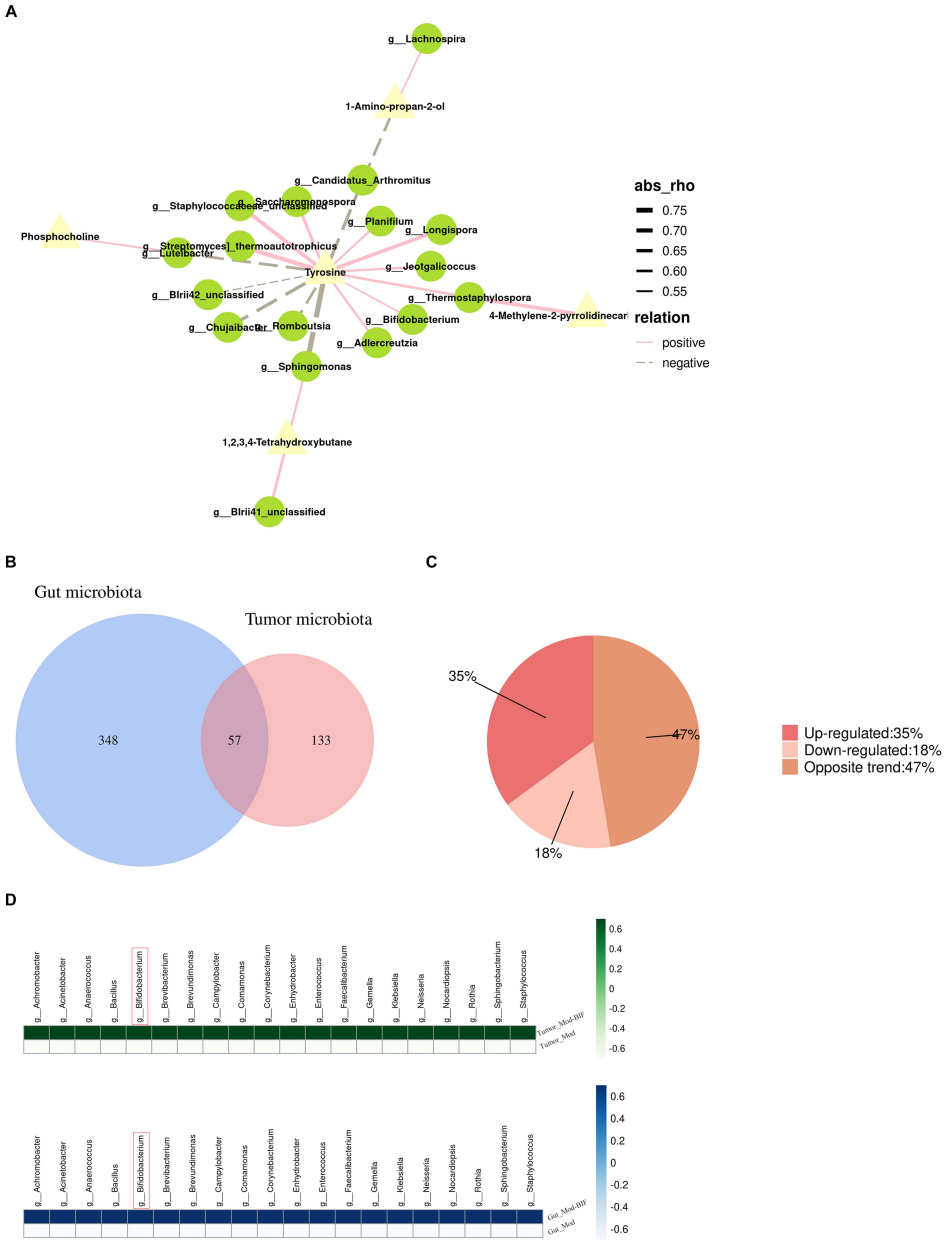
Relationship between gut microbiota and serum metabolites as well as the gut microbiota and tumor microbiota in glioma mice. **(A)** Network illustrating the correlation between differential metabolites and gut microbiota. **(B)** Venn diagram depicting the intersections between gut microbiota and tumor microbiota. **(C)** Pie chart of trends in total bacteria. **(D)** Heatmap of upregulated bacterial species common to both gut and tumor tissues. Mod, Model; BIF, *Bifidobacterium*.

By jointly analyzing differential microbiota and metabolites with a rho >0.5 and *p* < 0.05, as the threshold values indicated in Figure 5A and Supplementary Table S6, it is evident that tyrosine emerges as a crucial metabolite exhibiting strong correlations with most differential microbiota at the genus level. Furthermore, *Bifidobacterium*, which exhibited a significant increase following treatment, demonstrated a positive association with tyrosine.

To investigate the relationship between the abundance of gut microbiota and tumor microbiota, we conducted a genus-level analysis of all bacteria present in both groups. In both gut and tumor tissues, we identified 57 shared bacterial species (Figure 5B). Among these 57 species of bacteria, we observed that the abundance of 20 bacterial species was upregulated, the abundance of 10 bacterial species was downregulated, and the abundance of 27 species exhibited opposite changes (Figure 5C). Surprisingly, we found that *Bifidobacterium* exhibited increased abundance in both gut and tumor tissues (Figure 5D), suggesting a potential influence of alterations in gut microbiota on the composition of tumor microbiota.

### 3.7 *Bifidobacterium* suppressed MEK/ERK cascade and *Wnt5a* mRNA levels to inhibit glioma

**Figure 6:**
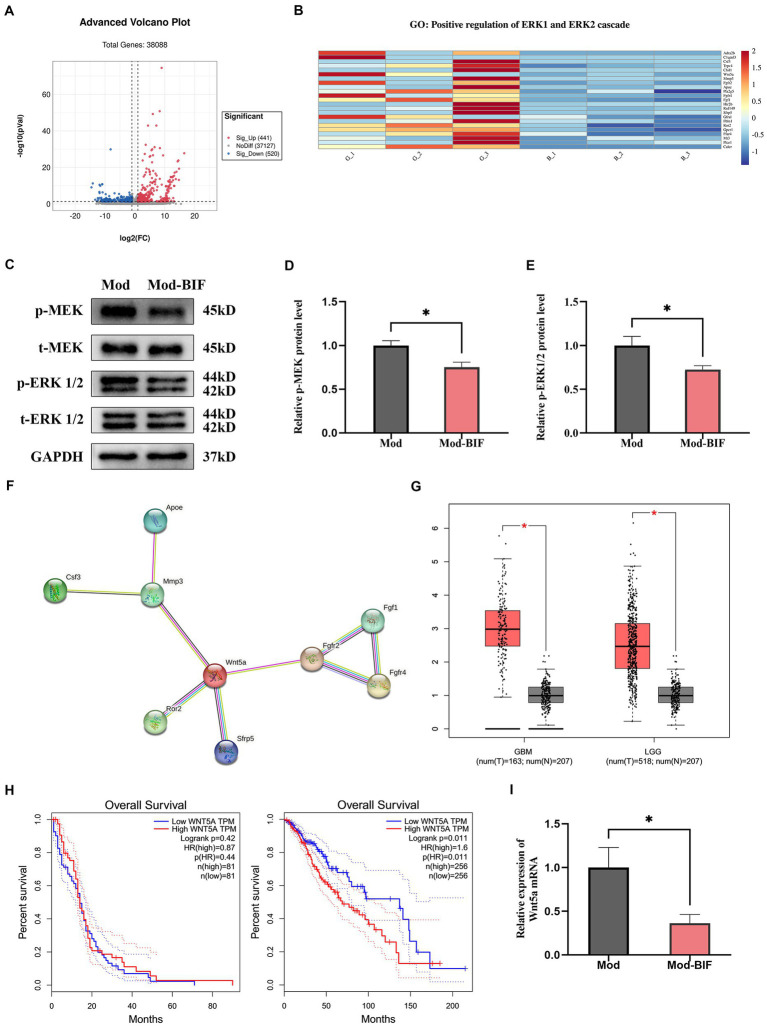
*Bifidobacterium* suppressed glioma through the inhibition of the MEK/ERK cascade and the downregulation of the Wnt5a mRNA level. **(A)** Volcano plot of DEGs in glioma tissues between the Mod and Mod-BIF groups (*n* = 3). **(B)** Heatmap displaying downregulated DEGs associated with “GO:0070374” in the Mod and Mod-BIF groups (*n* = 3). **(C)** Representative immunoblots for p-MEK, t-MEK, p-ERK 1/2, t-ERK 1/2 and quantitative analysis of **(D)** p-MEK and **(E)** p-ERK1/2 in glioma tissues (*n* = 6; independent sample *t*-test). **(F)** PPI network of downregulated DEGs enriched in “GO:0070374.” **(G)** Differential expression of Wnt5a in normal and glioma tissues corresponding to TCGA and GTEx datasets. **(H)** Kaplan–Meier overall survival curves of patients with glioma according to Wnt5a expression levels. **(I)** mRNA expression of Wnt5a in glioma tissues from the Mod and Mod-BIF groups (*n* = 6; independent sample *t*-test). Mod, Model; BIF, *Bifidobacterium*. Experimental data are expressed as the mean ± SEM, ^*^p < 0.05.

After initially establishing that *Bifidobacterium* could inhibit glioma, we proceeded to investigate changes in the glioma genes through transcriptome sequencing. Differentially expressed genes (DEGs) between the Mod and Mod-BIF groups were identified based on a significance level of *p* < 0.05 and |log2 fold change| ≥ 1. We found 441 upregulated and 520 downregulated DEGs (Figure 6A). Subsequently, we conducted a Gene Ontology (GO) enrichment analysis on the downregulated DEGs. This analysis revealed an enrichment of downregulated DEGs in the GO term 0070734, specifically related to the positive regulation of the ERK1 and ERK2 cascades. To visually represent the differences between these genes in the two groups, we conducted a heatmap analysis of the downregulated DEGs associated with this GO term (Figure 6B).

The MEK/ERK cascade plays a pivotal role in the mitogen-activated pathway, facilitating the transmission of growth signals from the cell surface to the nucleus. Furthermore, the MEK/ERK pathway modulates the transcription of numerous genes to bolster cell proliferation (Choudhury et al., 2014). Moreover, the activation of the MEK/ERK cascade is associated with the invasive behavior of glioma cells (Huang et al., 2023). Western blot analysis was employed to assess the protein levels of MEK and ERK. The results revealed a significant reduction in the phosphorylation levels of MEK and ERK1/2 proteins in glioma tissues upon treatment with *Bifidobacterium* (*p* < 0.05; Figures 6C–E). This suggests that *Bifidobacterium* may exert an inhibitory effect on MEK and ERK1/2 protein phosphorylation in glioma tissues, thereby suppressing the MEK/ERK cascade. Original western blot images are presented in Supplementary Figure S3.

Additionally, through a protein–protein interaction (PPI) network established via the STRING database for the analysis of interactions among the downregulated DEGs, we discerned that *Wnt5a* served as the central hub gene (Figure 6F). *Wnt5a*, a member of the WNT family, has been previously demonstrated to induce rapid glioma growth and migration while also being associated with the survival of tumor-associated microglia (Xu et al., 2020). Subsequently, we assessed the differential expression and prognostic significance of *Wnt5a* by utilizing the Cancer Genome Atlas (TCGA) and Genotype-Tissue Expression (GTEx) datasets through the Gene Expression Profiling Interactive Analysis platform. In both GBM and low-grade glioma (LGG), the expression of *Wnt5a* was significantly increased in glioma tissues compared with normal tissues (*p* < 0.05; Figure 6G). However, the expression level of *Wnt5a* was associated with overall survival only in LGG (Figure 6H). In the context of our study, qRT-PCR results demonstrated a reduction in the mRNA expression of *Wnt5a* as a consequence of *Bifidobacterium* treatment (Figure 6I).

## 4 Discussion

Numerous studies have demonstrated a close association between gut microbiota and various diseases, highlighting its impact on brain functions through the gut-brain axis (Mehrian-Shai et al., 2019). Therefore, in this study, we investigated the inhibitory effects of *Bifidobacterium* on glioma. Employing MRI, HE staining, and survival curves, we elucidated that *Bifidobacterium* exhibited the capacity to suppress tumor volume and extend the survival time of glioma mice. Further investigations showed that *Bifidobacterium* inhibits glioma without liver or kidney toxicity. Nejman et al. validated the presence of microbes in tumors, including glioma, and identified the presence of *Bifidobacteriales* (Nejman et al., 2020). In our study, we likewise identified the presence of *Bifidobacterium* in both groups. We found that the administration of *Bifidobacterium* led to alterations in the composition of microbes in glioma. In addition, in the GL261 glioma model, we conclusively confirmed that the predominant tumor microbiota primarily consists of *Proteobacteria*, *Firmicutes*, *Actinobacteria*, *Bacteroidetes*, and *Cyanobacteria* at the phylum level.

The mitogen-activated protein kinase (MAPK) family regulates gene transcription and expression, playing crucial roles in various biological processes such as cell growth, proliferation, apoptosis, and angiogenesis (Hsu et al., 2018). Among the key members of the MAPK family, ERK1/2 stands out as the exclusive known substrate of MEK1/2, and its abnormal activation is associated with a poor prognosis in cancer (Fu L. L. et al., 2022). The activation of ERK1/2 influences multiple substrates and exerts profound effects on various cellular processes (Lavoie et al., 2020). Additionally, the overactivation of ERK1/2 represents the most prevalent dysregulated kinase pathway in GBM cells, contributing to the proliferation, invasion, apoptosis, and stress response of these cells (Hannen et al., 2017). *Lactobacillus reuteri* can produce histamine, leading to the upregulation of cAMP, which subsequently suppresses downstream MEK/ERK signaling via protein kinase A, resulting in reduced TNF production and anti-inflammatory effects (Thomas et al., 2012). Moreover, microbiota-derived metabolites such as kynurenic acid, urolithin A and short-chain fatty acids (SCFAs) could suppress the phosphorylation of ERK1/2 to inhibit tumor progression (Yang et al., 2023). Additionally, oncology clinical trials targeting ERK1/2 are underway or have been completed (NCT06310382, NCT02857270, etc.) (*clinicaltrials.gov*). Combined with the results of RNA-Seq, we studied the MEK/ERK cascades. Our results showed that *Bifidobacterium* significantly reduces the phosphorylation levels of MEK1/2 and ERK1/2 proteins in gliomas. This suggests that *Bifidobacterium* can partially inhibit the transmission of the MEK/ERK cascade. When we found that *Bifidobacterium* could inhibit gliomas by modulating microbes, metabolites, and MEK–ERK cascades through multi-omics analyses, we wanted to further analyze the effects of *Bifidobacterium* on immune and inflammatory responses. Research showed that probiotics could strengthen the efficacy of Immune checkpoint inhibitors such as programmed cell death 1 (PD-1) /programmed cell death ligand 1 (PD-L1) with *Bifidobacterium* strengthening the efficacy of anit-PD-1/PD-L1 on melanoma-bearing mice (Miller and Carson, 2020). Therefore, we investigated that whether *Bifidobacterium* could regulate the expression of PD-1/PD-L1. Our findings showed that *Bifidobacterium* could decrease PD-1 expression on protein level and significantly suppress the protein expression of PD-L1 (*p* < 0.0001, Supplementary Figures S4A–C), indicating that *Bifidobacterium* might synergize with anti-PD-L1 to inhibit gliomas, but needing further investigation.

NF-κB is involved in inflammatory response and cancers. Activated NF-κB regulate various proinflammatory cytokines expression contributed to inflammatory response, however, aberrant activated NF-κB result in chronic inflammation and tumor development (Yu et al., 2020). We found that the expression of NF-κB of the Mod-BIF group was lower compared with the Mod group (*p* < 0.05, Supplementary Figures S4D,F), whereas there was not significance of the expression of p-NF-κB between the Mod and Mod-BIF groups (Supplementary Figures S4D,E).

Research findings have demonstrated the positive expression of LPS in several types of tumor tissues, while LTA has been primarily detected in melanoma and largely absent in other types of tumors (Nejman et al., 2020), consistent with our results. Moreover, due to low abundance of microbiota and potential external contamination of tumors, conventional microbial sequencing fails to yield accurate results (Nejman et al., 2020). Therefore, we employed 16S rDNA 5R sequencing to detect tumor microbiota, thereby significantly improving the coverage and resolution of microbial species detection and the accuracy of sequencing (Nejman et al., 2020). The abundance of tumor microbiota may serve as a more reliable prognostic indicator for LGG (Hermida et al., 2022). Current studies have suggested a relationship between tumor microbial diversity and the survival duration of patients with pancreatic cancer, with higher α-diversity observed in long-term survivors (Riquelme et al., 2019). In the context of our study, *Bifidobacterium* increased the α-diversity of tumor microbiota in glioma mice, potentially contributing to the prolonged survival time of these mice. Moreover, at the phylum level, we observed an upregulation of the relative abundance of *Actinobacteria* in the Mod-BIF group, similar to the findings of Erick et al. However, alterations in the relative abundance of *Proteobacteria* were contrary to these results (Riquelme et al., 2019). This might be due to the complex changes in host–microbe kinetics and microbe–microbe kinetics, which makes the causal relationship between microbes and tumors difficult to determine (Plottel and Blaser, 2011). For example, *Helicobacter* has been implicated in cancer, yet in some instances, *Helicobacter* has been shown to cooperate with symbionts to confer protective effects on the host (Sholl et al., 2022). Thus, further investigations are warranted to confirm the correlation between specific microbes and distinct tumor types.

Metabolites originating from the gut microbiota may mainly alter the tumor microenvironment and modulate important signaling pathways in cancer cells as well as various immune cells to affect cancer progression, and metabolites might interact with the central and enteric nervous systems through the gut–brain axis (Carabotti et al., 2015; Yang et al., 2023). Various prior studies have demonstrated an association between gut microbiota and CNS diseases, such as depression, anxiety, autism, Parkinson’s disease, and schizophrenia (Jaye et al., 2021). Our findings suggest that administration of *Bifidobacterium* significantly increases the abundance of *Bifidobacterium*, leading to the inhibition of glioma. Additionally, glioma can induce intestinal barrier damage in mice (Wang et al., 2022). However, we found no significant differential changes in occludin and ZO-1 proteins in the intestines of mice from the Mod and Mod-BIF groups. This suggests that *Bifidobacterium* may not ameliorate the intestinal barrier damage caused by glioma. Given that tumor microbiota may originate from intestinal microbes breaching the intestinal mucosa (Xie et al., 2022) and the permeability of the blood–brain barrier can be altered in high-grade glioma (Qiao et al., 2022), we speculated that a portion of the microbes in glioma may have originated from the gut microbiota in our study. Nevertheless, further investigations are necessary to substantiate this claim. Furthermore, although microbes in different tissues may appear to be independent, they could potentially be interconnected. Research has indicated that tumor microbiota might be influenced by gut microbiota via pancreatic duct communication in pancreatic cancer (Pushalkar et al., 2018; Aykut et al., 2019; Riquelme et al., 2019; Sepich-Poore et al., 2021). Here, we found that changes in the abundance of certain gut microbes may cause an increase or decrease in corresponding microbes within tumor tissues, although the exact mechanism remains unknown. Collectively, we speculate that there might be cross-body interactions facilitated by “microbe–microbe” interactions to some extent.

SCFAs, could promote cell apoptosis by inhibiting PI3K-Akt signaling in colon cancer cells, and kynurenic acid decreased the phosphorylation of Akt with suppressing PI3K-Akt–mTOR pathway to prevent the growth of colon cancer cells (Walczak et al., 2014; Ma et al., 2019). In addition, inhibition of Hippo/YAP signaling, upregulation of E-cadherin expression, and prevention of the formation of the invasive phenotype of cancer cells were caused by SCFAs binding to FFAR2 on the surface of breast cancer cells (Thirunavukkarasan et al., 2017). By blocking the Wnt/β-catenin signaling pathway, urolithin B suppressed hepatocellular carcinoma cells proliferation and growth and instead caused cell cycle arrest and apoptosis (Lv et al., 2019). Meanwhile, metabolites might influence the efficacy of chemotherapy and the effectiveness of immunotherapy by modulating immune activity in cancer patients (Yang et al., 2023). Therefore, the potential of metabolites in cancer therapy is promising. In our study, tyrosine emerged as a potential critical metabolite. Tyrosine is an aromatic amino acid essential for protein synthesis in living organisms and serves as an alternative energy source for molecular functions. Patients with esophageal cancer exhibit significantly reduced serum levels of tyrosine, which is enzymatically converted to phenol by β-tyrosinase, suggesting its potential as a marker for gastroesophageal cancer (Wiggins et al., 2015). Furthermore, tyrosine is a significant neurotransmitter-associated metabolite linked to brain function, potentially mediating the influence of the gut microbiota on smoking behavior (Fan et al., 2023). Additionally, 1,2,3,4-tetrahydroxybutane, also known as erythritol, is commonly employed as a “zero-calorie” or “non-nutritive” sugar substitute. However, recent research has raised concerns about its potential correlation with an increased risk of major adverse cardiovascular events and a propensity to foster enhanced thrombosis (Witkowski et al., 2023). Our results reveal that *Bifidobacterium* can modulate the levels of serum metabolites in glioma mice, and this modulation may be associated with alterations in the composition of the gut microbiota. Moreover, since metabolites such as tyrosine can be further metabolized by certain microorganisms, it is plausible that serum metabolites may penetrate glioma tissue, subsequently interacting with tumor-associated microbes.

Preparations of inanimate microorganisms or their constituents that are advantageous to the host’s health are known as postbiotics. The principal constituents of postbiotics include microbiota metabolites and bacterial components (such as exopolysaccharides, cell-free supernatants and enzymes, etc.) (Xie et al., 2024). Exopolysaccharides, bacterial surface macromolecules, could promote colon cancer cell apoptosis by regulating immune response; the cell-free supernatant which is biologically active metabolites secreted by microorganisms showed antimicrobial, antioxidant, antitumor activities, and induced apoptosis of colorectal cancer cells *in vitro*; enzymes encoded by microbes showed the abilities to inhibit chemicals such as AOM, DMH, TNBS induced colon cancer in animal models (Song et al., 2023). Therefore, in addition to microbes being a promising direction for glioma treatment, microbes-derived postbiotics might also be promising with further research being needed.

## 5 Conclusion

Taken together, our findings indicate that *Bifidobacterium* inhibits glioma development, in part by suppressing the MEK/ERK cascade as well as by influencing both tumor and gut microbiota. Thus, *Bifidobacterium* emerges as a promising candidate for glioma treatment.

## Data availability statement

The original contributions presented in the study are publicly available. This data can be found in the MetaboLights repository (https://www.ebi.ac.uk/metabolights/), accession number MTBLS8811.

## Ethics statement

The animal study was approved by the ethics committee of Experimental Animal Administration of Sichuan University (No. K2023008). The study was conducted in accordance with the local legislation and institutional requirements.

## Author contributions

HF: Conceptualization, Formal analysis, Investigation, Methodology, Writing – original draft, Writing – review & editing. YW: Investigation, Validation, Writing – review & editing. MH: Investigation, Validation, Writing – review & editing. LW: Data curation, Methodology, Writing – review & editing. XL: Validation, Writing – review & editing. XK: Supervision, Writing – review & editing. JD: Conceptualization, Supervision, Writing – review & editing. FP: Conceptualization, Funding acquisition, Project administration, Resources, Writing – review & editing.
