# Supplementary material for: Multi-omics-based investigation of Bifidobacterium’s inhibitory effect on glioma: regulation of tumor and gut microbiota, and MEK/ERK cascade
Source: Front Microbiol. 2024 Apr 18;15:1344284. doi: 10.3389/fmicb.2024.1344284 (PMC11064926; doi:10.3389/fmicb.2024.1344284)
Supplement: Supplementary file 1 [file Data_Sheet_1.PDF]

Supplementary Materials

Table S1. The microbial number of BI516 and LP-Onlly freeze-dried powder

Freeze-dried powder	Test Item	Microbial number	Test Method
BBR-15	*Bifidobacterium breve*	5.2×10^9 CFU/g	GB 4789.35-2016
JBLC-141	*Bifidobacterium longum*	2.1×10^9 CFU/g	GB 4789.35-2016
JYBR-190	*Bifidobacterium lactis*	6.9×10^9 CFU/g	GB 4789.35-2016
JYBB-163	*Bifidobacterium bifidum*	8.2×10^9 CFU/g	GB 4789.35-2016

Table S2. Physiological and biochemical characteristics of BBR-15

Control	Glycerin	Erythritol	D-xylose	L-Xylose	Adonol	Galactose
-	-	-	+	-	-	+
Glucose	Fructose	Mannose	Sorbose	Rhamnopyranose	Dulcitol	Inositol
+	+	-	-	-	-	-
Mannitol	Sorbitol	α-D-Methylglucoside	N-acetylglucosamine	Amygdalin	Esculin	Salicyl alcohol
-	-	-	-	-	-	-
Cellobiose	Maltose	Lactose	Melibiose	Sucrose	Trehalose	Inulin
-	+	+	-	+	-	-
Melezitose	Raffinose	Amylum	Glycogen	Gentian disaccharide	Gluconate	
-	-	-	-	+	-	

Note: "+" means positive reaction, "-" means negative reaction

Table S3. Physiological and biochemical characteristics of JBLC-141

Control	L-Arabinose	Ribose	D-xylose	L-Xylose	Adonol	Galactose
-	+	-	+	-	-	+
Glucose	Fructose	Mannose	Sorbose	Rhamnopyranose	Dulcitol	Inositol
+	+	-	-	-	-	-
Mannitol	Sorbitol	α-D-Methylglucoside	N-acetylglucosamine	Amygdalin	Esculin	Salicyl alcohol
-	-	-	-	-	-	-
Cellobiose	Maltose	Lactose	Melibiose	Sucrose	Trehalose	Inulin

-	+	+	-	+	-	-
Melezitose	Raffinose	Amylum	Glycogen	Gentian disaccharide	Gluconate	
+	+	-	-	-	-	

Note: "+" means positive reaction, "-" means negative reaction

Table S4. Physiological and biochemical characteristics of JYBR-190

Control	L-Arabinose	Ribose	D-xylose	L-Xylose	Adonol	Galactose
-	+	-	+	-	-	-
Glucose	Fructose	Mannose	Sorbose	Rhamnopyranose	Dulcitol	Inositol
+	-	-	-	-	-	-
Mannitol	Sorbitol	α-D-Methylglucoside	N-acetylglucosamine	Amygdalin	Esculin	Salicyl alcohol
+	-	-	-	+	-	+
Cellobiose	Maltose	Lactose	Melibiose	Sucrose	Trehalose	Inulin
-	+	+	+	+	-	-
Melezitose	Raffinose	Amylum	Glycogen	Gentian disaccharide	Gluconate	
-	-	-	-	-	-	

Note: "+" means positive reaction, "-" means negative reaction

Table S5. Physiological and biochemical characteristics of JYBB-163

Control	L-Arabinose	Ribose	D-xylose	L-Xylose	Adonol	Galactose
-	-	-	+	-	-	-
Glucose	Fructose	Mannose	Sorbose	Rhamnopyranose	Dulcitol	Inositol
+	-	-	-	-	-	-
Mannitol	Sorbitol	α-D-Methylglucoside	N-acetylglucosamine	Amygdalin	Esculin	Salicyl alcohol
-	-	-	-	-	-	-
Cellobiose	Maltose	Lactose	Melibiose	Sucrose	Trehalose	Inulin
-	+	+	-	+	-	-
Melezitose	Raffinose	Amylum	Glycogen	Gentian disaccharide	Gluconate	
-	-	-	-	+	-	

Note: "+" means positive reaction, "-" means negative reaction

Table S6. The R Code used for figures

Content	R code
Venn	R-3.4.4
NMDS	R-3.4.4
α-diversity	R-3.4.4
Circos	R-3.4.4
LEfSe	Nsegata-lefse
Bubble diagram	R-3.4.4
Manhattan	R-3.4.4
Heatmap	R-3.4.4
Correlated Network	R-3.4.4
Picrust2	Picrust2
Volcano plot	R-3.4.4

Table S6. The Spearman analysis of differential microbiota and metabolites

Genus	Metabolite	Rho	*P* value	Relation
Adlercreutzia	Tyrosine	0.57	0.02	Positive
Bifidobacterium		0.52	0.04	Positive
BIrii42_unclassified		-0.50	0.047	Negative
Candidatus_Arthromitus		-0.63	0.01	Negative
Chujaibacter		-0.64	0.01	Negative
Jeotgalicoccus		0.57	0.02	Positive
Longispora		0.66	0.01	Positive
Luteibacter		-0.60	0.01	Negative
Planifilum		0.55	0.03	Positive
Romboutsia		-0.60	0.01	Negative
Saccharomonospora		0.62	0.01	Positive
Sphingomonas		-0.76	0.001	Negative
Staphylococcaceae_unclassified		0.68	0.004	Positive
Streptomyces]_thermoautotrophicus		0.70	0.002	Positive
Thermostaphylospora		0.58	0.02	Positive
BIrii41_unclassified	1,2,3,4-Tetrahydroxybutane	0.60	0.01	Positive
Sphingomonas		0.56	0.02	Positive
Candidatus_Arthromitus	1-Amino-propan-2-ol	-0.60	0.01	Negative
Lachnospira		0.53	0.03	Positive
Luteibacter	Phosphocholine	0.54	0.03	Positive
Thermostaphylospora	4-Methylene-2-pyrrolidinecarboxylic acid	0.66	0.01	Positive

Sequence S1. 16S rDNA gene sequence of BBR-15

CTGGCGGCGTGCTTAACACATGCAAGTCGAACGGGATCCATCGGGCTTTGCTTGGTGGTGAGAGTGGC
GAACGGGTGAGTAATGCGTGACCGACCTGCCCCATGCACCGGAATAGCTCCTGGAAACGGGTGGTAAT
GCCGGATGCTCCATCACACCGCATGGTGTGTTGGGAAAGCCTTTGCGGCATGGGATGGGGTCGCGTCCT
ATCAGCTTGATGGCGGGGTAACGGCCCACCATGGCTTCGACGGGTAGCCGGCCTGAGAGGGCGACCGG
CCACATTGGGACTGAGATACGGCCCAGACTCCTACGGGAGGCAGCAGTGGGGAATATTGCACAATGGG
CGCAAGCCTGATGCAGCGACGCCGCGTGAGGGATGGAGGCCTTCGGGTTGTAAACCTCTTTTGTTAGG
GAGCAAGGCACTTTGTGTTGAGTGTACCTTTCGAATAAGCACCGGCTAACTACGTGCCAGCAGCCGCGG
TAATACGTAGGGTGCAAGCGTTATCCGGAATTATTGGGCGTAAAGGGCTCGTAGGCGGTTCGTCGCGTC
CGGTGTGAAAGTCCATCGCTTAACGGTGGATCCGCGCCGGGTACGGGCGGGCTTGAGTGCGGTAGGGG
AGACTGGAATTCCCGGTGTAACGGTGGAATGTGTAGATATCGGGAAGAACACCAATGGCGAAGGCAGG
TCTCTGGGCCGTTACTGACGCTGAGGAGCGAAAGCGTGGGAGCG

Sequence S2. 16S rDNA gene sequence of JBLC-141

GCTGGCGGCGTGCTTAACACATGCAAGTCGAACGGGATCCATCAAGCTTGCTTGGTGGTGAGAGTGGC
GAACGGGTGAGTAATGCGTGACCGACCTGCCCCATACACCGGAATAGCTCCTGGAAACGGGTGGTAAT
GCCGGATGCTCCAGTTGATCGCATGGTCTTCTGGGAAAGCTTTCGCGGTATGGGATGGGGTCGCGTCCTA
TCAGCTTGACGGCGGGGTAACGGCCCACCGTGGCTTCGACGGGTAGCCGGCCTGAGAGGGCGACCGG
CCACATTGGGACTGAGATACGGCCCAGACTCCTACGGGAGGCAGCAGTGGGGAATATTGCACAATGGG
CGCAAGCCTGATGCAGCGACGCCGCGTGAGGGATGGAGGCCTTCGGGTTGTAAACCTCTTTTATCGGGG
AGCAAGCGAGAGTGAGTTTACCCGTTGAATAAGCACCGGCTAACTACGTGCCAGCAGCCGCGGTAATA
CGTAGGGTGCAAGCGTTATCCGGAATTATTGGGCGTAAAGGGCTCGTAGGCGGTTCGTCGCGTCCGGTG
TGAAAGTCCATCGCTTAACGGTGGATCCGCGCCGGGTACGGGCGGGCTTGAGTGCGGTAGGGGAGACT
GGAATTCCCGGTGTAACGGTGGAATGTGTAGATATCGGGAAGAACACCAATGGCGAAGGCAGGTCTCT
GGGCCGTTACTGACGCTGAGGAGCGAAAGCGTGGGGA

Sequence S3. 16S rDNA gene sequence of JYBR-190

GCTGGCGGCGTGCTTAACACATGCAAGTCGAACGGGATCCCTGGCAGCTTGCTGTCGGGGTGAGAGTG
GCGAACGGGTGAGTAATGCGTGACCAACCTGCCCTGTGCACCGGAATAGCTCCTGGAAACGGGTGGTA
ATACCGGATGCTCCGCTCCATCGCATGGTGGGGTGGGAAATGCTTTTGCGGCATGGGATGGGGTCGCGT
CCTATCAGCTTGTTGGCGGGGTGATGGCCCACCAAGGCGTTGACGGGTAGCCGGCCTGAGAGGGTGAC
CGGCCACATTGGGACTGAGATACGGCCCAGACTCCTACGGGAGGCAGCAGTGGGGAATATTGCACAAT
GGGCGCAAGCCTGATGCAGCGACGCCGCGTGCGGGATGGAGGCCTTCGGGTTGTAAACCGCTTTTGTT
CAAGGGCAAGGCACGGTTTCGGCCGTGTTGAGTGGATTGTTCGAATAAGCACCGGCTAACTACGTGCC
AGCAGCCGCGGTAATACGTAGGGTGCGAGCGTTATCCGGATTTATTGGGCGTAAAGGGCTCGTAGGCGG
TTCGTCGCGTCCGGTGTGAAAGTCCATCGCCTAACGGTGGATCTGCGCCGGGTACGGGCGGGCTGGAG
TGCGGTAGGGGAGACTGGAATTCCCGGTGTAACGGTGGAATGTGTAGATATCGGGAAGAACACCAATG
GCGAAGGCAGGTCTCTGGGCCGTCACTGACGCTGAGGAGCGAAAGC

Sequence S4. 16S rDNA gene sequence of JYBB-163

GGATGAACGCTGGCGGCGTGCTTAACACATGCAAGTCGAACGGGATCCATCAAGCTTGCTTGGTGGTG
AGAGTGGCGAACGGGTGAGTAATGCGTGACCGACCTGCCCCATGCTCCGGAATAGCTCCTGGAAACGG
GTGGTAATGCCGGATGTTCCACATGATCGCATGTGATTGTGGGAAAGATTCTATCGGCGTGGGATGGGGT
CGCGTCCTATCAGCTTGTTGGTGAGGTAACGGCTCACCAAGGCTTCGACGGGTAGCCGGCCTGAGAGG
GCGACCGGCCACATTGGGACTGAGATACGGCCCAGACTCCTACGGGAGGCAGCAGTGGGGAATATTGC
ACAATGGGCGCAAGCCTGATGCAGCGACGCCGCGTGAGGGATGGAGGCCTTCGGGTTGTAAACCTCTT
TTGTTTGGGAGCAAGCCTTCGGGTGAGTGTACCTTTCGAA
TAAGCGCCGGCTAACTACGTGCCAGCAGCCGCGGTAATACGTAGGGCGCAAGCGTTATCCGGATTTATT
GGGCGTAAAGGGCTCGTAGGCGGCTCGTCGCGTCCGGTGTGAAAGTCCATCGCTTAACGGTGGATCTG
CGCCGGGTACGGGCGGGCTGGAGTGCGGTAGGGGAGACTGGAATTCCCGGTGTAACGGTGGAATGTGT
AGATATCGGGAAGAACACCGATGGCGAAGGCAGGTCTCTGGGCCGTCACTGACGCTGAGGAGCGAAA
GC

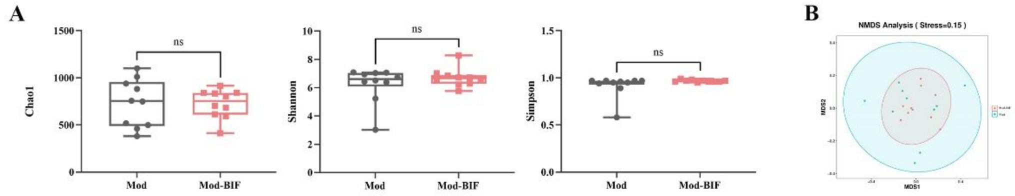

Figure S1. Comparsion of (**A**) α-diversity and (**B**) NMDS analysis based on binary-jaccard distance of gut microbiota between the Mod and Mod-BIF groups. Mod: Model, BIF: *Bifidobacterium*. Experimental data are expressed as Mean ± SEM, n=10.

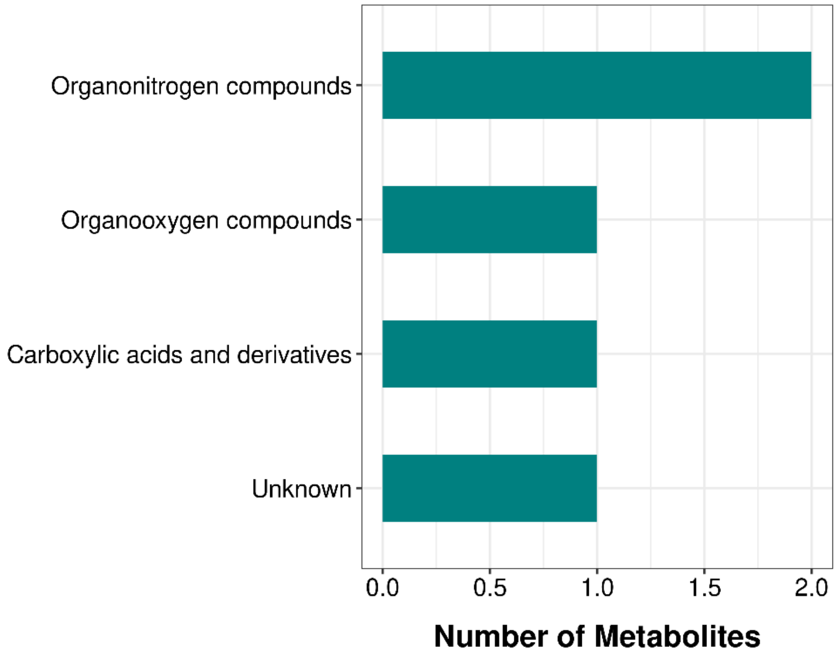

Figure S2. HMDB classification map of differential metabolites.

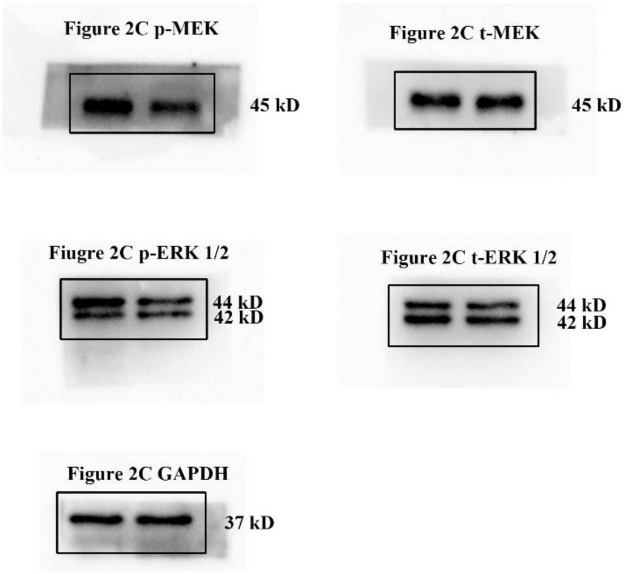

Figure S3. Original images of Western blot. The bands from left to right respectively represent Mod and Mod-BIF group. Mod: Model, BIF: *Bifidobacterium*.

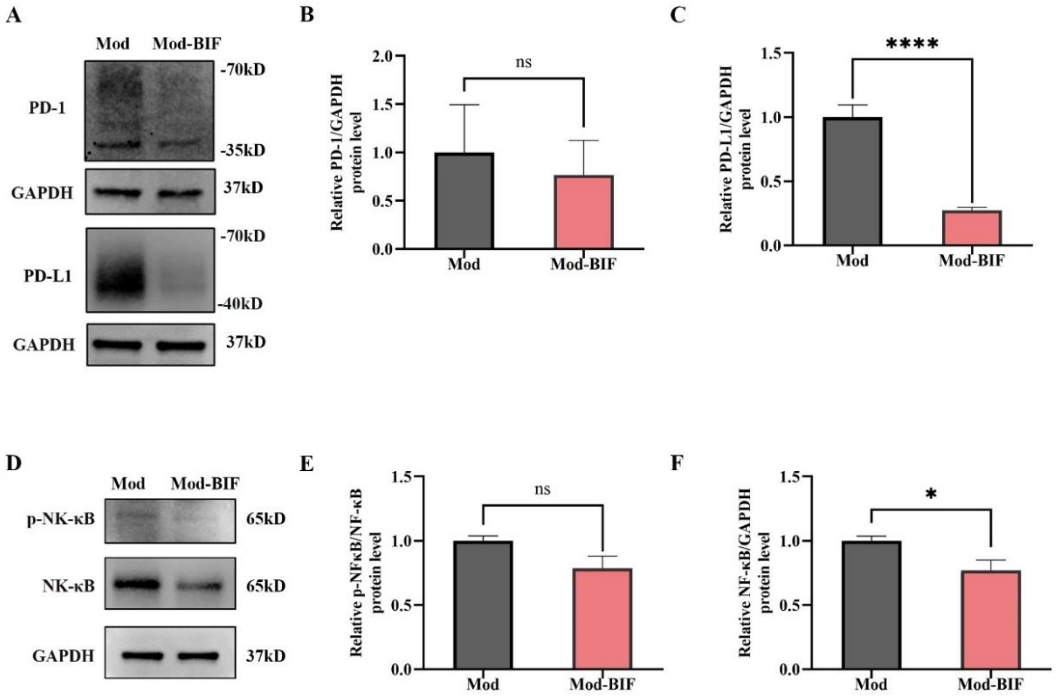

Figure S4. *Bifidobacterium* decreased the protein expressions of PD-1, PD-L1, p-NF-κB and NF-κB. (A) Representative immunoblots for PD-1, PD-L1 and quantitative analysis of (B) PD-1 and (C) PD-L1 in glioma tissues (n = 6; independent sample t-test). (D) Representative immunoblots for p-NF-κB, NF-κB and quantitative analysis

of (E) p-NF-κB and (F) NF-κB in glioma tissues (n = 6; independent sample t-test). Mod: Model, BIF: *Bifidobacterium*. Experimental data are expressed as the mean ± SEM, $^{*}P < 0.05$, $^{****}P < 0.0001$.

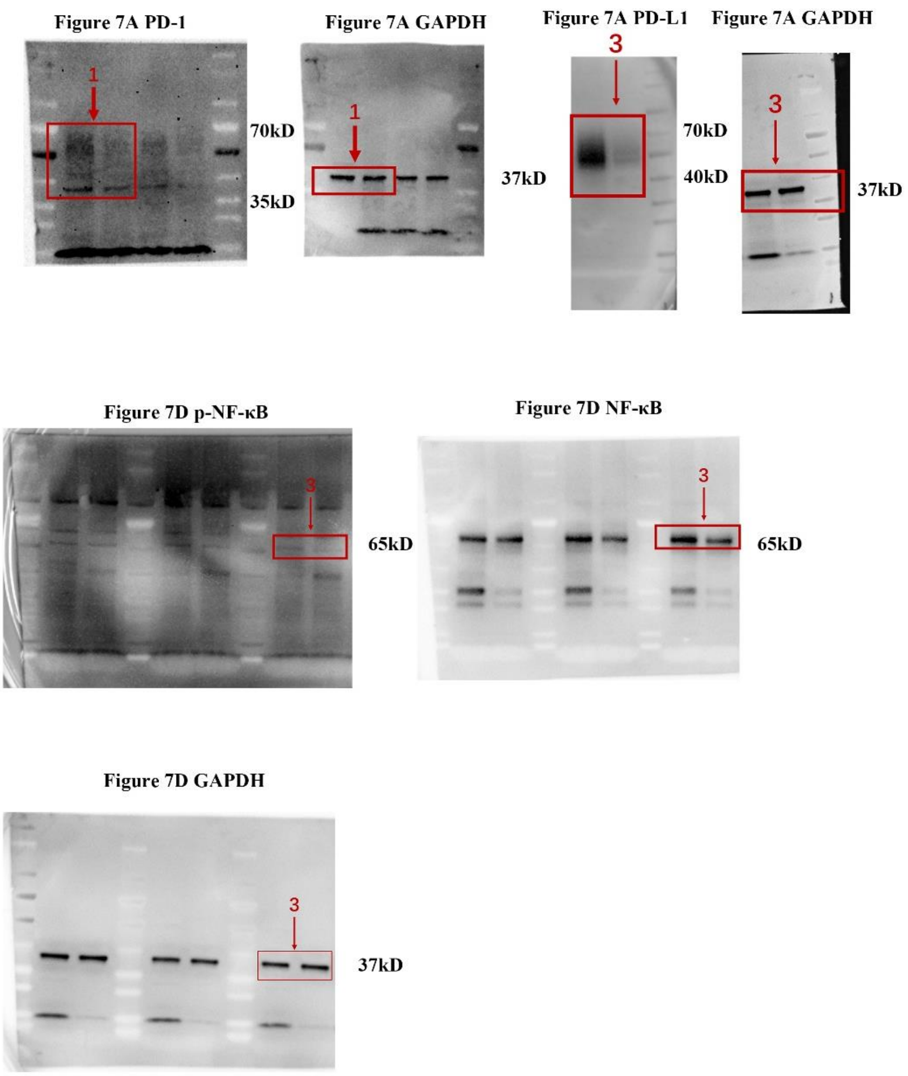

Figure S5. Original images of Western blot. The bands from left to right respectively represent Mod and Mod-BIF group.
